# The Bitter Gourd Transcription Factor *McNAC087* Confers Cold Resistance in Transgenic Arabidopsis

**DOI:** 10.3390/plants14223440

**Published:** 2025-11-10

**Authors:** Xuetong Yang, Kai Wang, Feng Guan, Bo Shi, Yuanyuan Xie, Chang Du, Tong Tang, Zheng Yang, Shijie Ma, Xinjian Wan

**Affiliations:** 1 Institute of Vegetables and Flowers, Jiangxi Academy of Agricultural Sciences, Nanchang 330200, China; yangxuetong@jxaas.cn (X.Y.); wangkai@jxaas.cn (K.W.); guanfeng_0813@163.com (F.G.); shibo_jiangxi@163.com (B.S.); m18073933758@163.com (Y.X.); 2 Jiangxi Key Laboratory of Horticultural Crops (Fruit, Vegetable & Tea) Breeding, Jiangxi Academy of Agricultural Sciences, Nanchang 330200, China; 3 Jiangxi Engineering Research Center of Vegetable Molecular Breeding, Jiangxi Academy of Agricultural Sciences, Nanchang 330200, China; 4 Guangdong Provincial Key Laboratory of Biotechnology for Plant Development, School of Life Science, South China Normal University, Guangzhou 510631, China; duchang@m.scnu.edu.cn; 5 Department of Computer Science and Information Technologies, Elviña Campus, University of A Coruña, 15001 A Coruña, Spain; tongtang@scu.edu.cn; 6 Zhengzhou Research Base, State Key Laboratory of Cotton Bio-Breeding and Integrated Utilization, School of Agricultural Sciences, Zhengzhou University, Zhengzhou 450001, China; yangz@zzu.edu.cn; 7 Crop Research Institute, Anhui Academy of Agricultural Sciences, Hefei 230031, China; mashijie@nwafu.edu.cn

**Keywords:** bitter gourd, *McNAC087*, overexpression, cold stress, physiological regulation

## Abstract

Low-temperature stress severely restricts the growth, development, and yield of bitter gourd *(Momordica charantia* L.), a warm-loving crop with inherent low cold tolerance. *NAC* transcription factors (TFs) serve as crucial regulators in plant responses to abiotic stresses like cold, while their roles in coping with cold stress in bitter gourd remain unclear. This study identified cold-responsive genes in bitter gourd and characterized the candidate *NAC* TF *McNAC087* through transcriptome analysis. Transcriptome sequencing of cold-tolerant (R) and cold-sensitive (S) bitter gourd inbred lines under 5 °C stress (0 h, 6 h, 12 h, 24 h) revealed 1157 co-expressed differentially expressed genes (DEGs), enriched via Kyoto Encyclopedia of Genes and Genomes (KEGG) analysis in cold tolerance-related pathways (signal transduction, carbohydrate/amino acid metabolism). RT-qPCR showed higher *McNAC087* expression in R than S under cold stress, and subcellular localization confirmed it as a nucleus-localized protein. *McNAC087* overexpression in Arabidopsis enhanced cold tolerance after sequential stress (−14 °C for 1.5 h, 4 °C for 16 h, and 22 °C recovery for 2 days), with less damage compared to wildtype (WT). Physiologically, overexpressing lines had higher proline, elevated superoxide dismutase/peroxidase/catalase (SOD/POD/CAT) activities, lower malondialdehyde/hydrogen peroxide/superoxide anion (MDA/H_2_O_2_/O_2_^−^) accumulation under cold stress, and upregulated *ICE*-*CBF*-*COR* pathway marker genes (*CBF1*, *DREB2A*, *RD29A*, *COR47*). In conclusion, *McNAC087* enhances Arabidopsis cold tolerance by regulating physiology and activating cold-responsive genes, providing insights for bitter gourd cold tolerance mechanisms and crop breeding.

## 1. Introduction

Temperature is a crucial factor in accurately assessing the geographical distribution of plants, functioning as the pre-eminent limiting factor [1]. Low temperatures pose a significant challenge, hindering plant growth, delaying development, and compromising yield potential. When exposed to low-temperature stress, plants experience alterations in their molecular regulatory networks, enzyme activities, various osmoregulatory substances, and cell structures [2]. At the stage of seed germination, low temperature inhibits enzyme activity in the seed, hinders water absorption, reduces the germination rate and prolongs the germination time [3]. At the seedling stage, low temperature leads to slow-growing, abnormal leaf development, and the inhibition of photosynthesis. At the flowering and fruiting stages, low temperature affects flower bud differentiation, pollen viability, pollination and fertilization, resulting in flower and fruit drop, poor fruit development, and a significant decline in yield and quality. Under conditions of low-temperature stress, younger tissues and organs sustain more severe damage. Moreover, the sensitivity of plants to low temperatures is heightened during the reproductive stage in comparison to the vegetative stage [4].

The transcriptional regulation of genes represents one of the most critical mechanisms that underlies plant resistance to harmful environments [5]. The transcription factor family exerts an essential role in coping with cold stress by modulating the levels of cold-related genes through either activation or repression processes in plants. The *CBF* transcription factor (*CBF*) family has been intensively discussed as a master regulator governing the response to cold stress in the plant kingdom. The *ICE*-*CBF*-*COR* signaling pathway is the most extensively investigated molecular regulatory mechanism governing plant responses to low-temperature conditions [6]. When subjected to low temperatures that do not cause freezing, plants can swiftly induce and upregulate the expression of *CBF* genes within 15 min, which is subsequently followed by the triggering of downstream cold-responsive (*COR*) genes. At present, CBF homologous genes have been characterized in a range of crops, encompassing rice, tomato, maize, wheat, and barley. Most of these genes are induced by low temperatures and regulate plant tolerance to low temperatures [1,7]. In addition, improved cold tolerance can be achieved through *CBF*-independent pathway, for example, the *SD6* (encoding a *bHLH* transcription factor)-*ICE2* molecular module senses ambient temperature to regulate seed dormancy, under normal temperature, the *SD6* gene is highly expressed, and *ICE2* gene expression is significantly inhibited to promote seed germination. At low temperature, the expression of the *SD6* gene was significantly inhibited, and the expression of the *ICE2* gene was upregulated so that the seeds remained dormant [8]. *NAC* TF serve as pivotal plant-specific regulators in plant development, stress responses, and metabolic pathways, exerting multifaceted functions across diverse biological processes throughout the entire plant life cycle [9,10]. *NACs* modulate cell differentiation, organogenesis, and developmental phase transitions via the regulation of specific target gene expression [11]. *NACs* participate in mediating plant tolerance to cold stress [3]. For instance, *SlNAC3*, which belongs to the NAC transcription factor family in tomato, plays a role in promoting the early adaptive response under 4 °C low-temperature stress [12]. *OsNAC5* exerts a positive regulatory effect on cold tolerance by maintaining the balance between ABA and ROS signaling [13]. By engaging in the *CBF*–*COR* pathway, *GmNAC20* contributes to the regulation of cold tolerance in soybean [14]. *MaICE1* targets the *MaNAC1* gene in banana, and the *MaNAC1* protein engages in interactions with *MaCBF1* to regulate the ability of banana fruit to tolerate cold stress [15].

Bitter gourd has distributed large-scale cultivation around the world except for cold regions. Owing to their unique bitter taste characteristics and diverse application values, bitter gourd plays an irreplaceable role in numerous fields, including food processing, pharmaceutical research and development, and agricultural production. Bitter gourd is a warm-loving crop with heat tolerance, yet its cold tolerance is extremely limited. As a crucial environmental limiting factor, low-temperature stress severely hinders the growth process, physiological metabolism, and morphogenesis of bitter gourd especially in the early spring and late autumn. In-depth analysis of the key genes involved in low-temperature tolerance and the molecular regulatory mechanism of bitter gourd can lay a solid theoretical foundation, provide a technical path for genetic improvement, and significantly accelerate the breeding process of new varieties of bitter gourd with low-temperature tolerance to obtain excellent varieties that can adapt to complex environments. However, there is no available information on how *NAC* transcription factors regulate plant resistance to low temperature.

The focus of this study was to determine the key genes that respond to low-temperature stress in bitter gourd. According to the transcriptome data analysis of cold-tolerant and cold-sensitive materials subjected to low-temperature stress for 0 h, 6 h, 12 h and 24 h, gene expression measurements and gene overexpression verification revealed that the expression of the transcription factor *McNAC087* in bitter gourd was closely related to how plants respond to low-temperature stress, which offers a theoretical basis for the genetic improvement of bitter gourd.

## 2. Results

### 2.1. Morphological Responses of Momordica charantia L. Under Low-Temperature Stress

**Figure 1:**
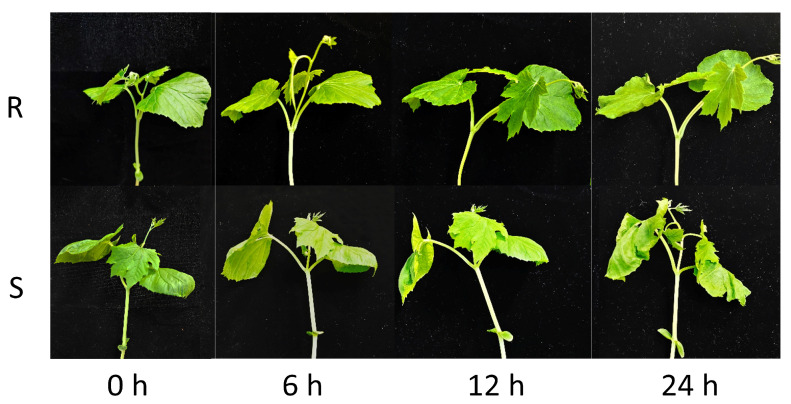
Phenotypic observations of cold-tolerant (R) and cold-sensitive (S) bitter gourd under cold exposure treatment for 0, 6, 12, and 24 h. Seedlings at the four-leaf and one-heart stage were treated with 5 °C low temperature.

Under low-temperature stress, bitter gourd seedling growth was significantly inhibited. Cold-tolerant inbred line (R) and cold-sensitive inbred line (S) seedlings underwent 5 °C exposure for 0, 6, 12, and 24 h, and different low-temperature response reactions were observed as the low-temperature treatment time increased. The most significant differences in seedling morphology were observed at 24 h of low-temperature treatment, with S seedlings exhibiting typical damage characteristics, including leaf wilting, drooping, curling, and dehydration. However, R is not sensitive to low-temperature treatment, and there were no obvious changes in leaf morphology (Figure 1).

### 2.2. RNA Sequencing

To identify and characterize the transcriptome of the bitter gourd seedlings under cold stress, we sequenced the R and S leaves treated at 5 °C for 0, 6, 12, and 24 h. Each treatment and each stage were repeated in triplicate, resulting in the construction of 24 cDNA libraries. The total number of raw reads ranged from 37,975,582 to 48,058,064, and the percentage of clean reads was greater than 99.51%. The percentage of clean reads with a Q30-based percentage (Phred value > 30) was greater than 96.71%, with a GC content of 46.37–54.02% (Table 1). The results indicated that the quality of the transcriptome sequencing data was highly reliable and that in-depth analysis could be conducted.

### 2.3. Sample Analysis

**Figure 2:**
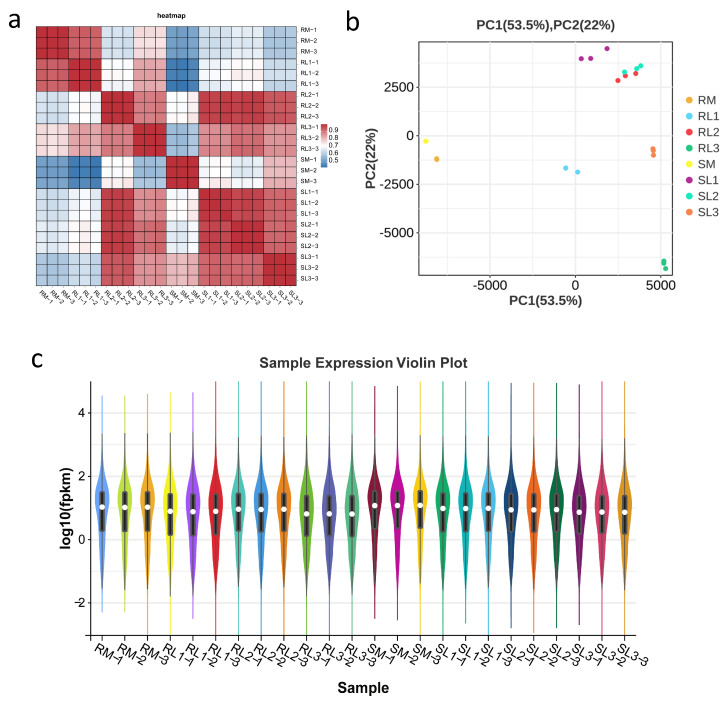
Transcriptome analysis of bitter gourd cultivars under cold stress. (**a**) Pearson’s correlation coefficient of bitter gourd under cold stress. (**b**) PCA of the transcriptome data of bitter gourd under cold stress. (**c**) Violin plot of bitter gourd expression.

The Pearson correlation coefficient (r) was utilized to represent the correlation between gene expression levels among samples. The closer r is to 1, the more similar the expression patterns are between samples. As shown in Figure 2a, the three replicate samples strongly correlated, with r values close to 1. The results of principal component analysis (PCA) revealed that the variance explained by the two principal components was 53.5% and 22%. The distribution of sample scores on these two principal components was clearly separated, indicating that there were distinguishable differences between sample populations according to PCA (Figure 2b). The violin plot compares the differences in the distributions of the different groups and shows the distributions of the R and S samples with different stress durations (Figure 2c).

### 2.4. Statistical Analysis of the Differentially Expressed Gene (DEG)

**Figure 3:**
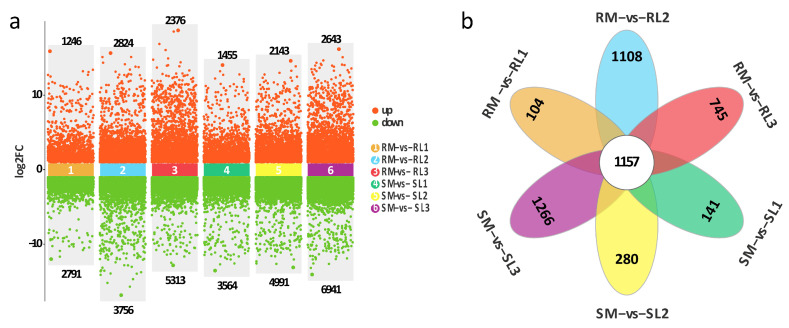
Identification of DEGs among two bitter gourd cultivars under cold stress. (**a**) Volcano plots of DEGs among different comparison groups. (**b**) Venn diagram of DEGs among different comparison groups.

DEGs were identified according to the screening criteria FDR < 0.05 and |log2 (fold change)| > 2, and the statistics of the number of DEGs under different cultivars (R and S) and low-temperature treatment times are shown in Figure 3a. There were 4037 (1246 upregulated, 2791 downregulated), 6580 (2824 upregulated, 3756 downregulated), 7689 (2376 upregulated, 5313 downregulated), 5019 (1455 upregulated, 3564 downregulated), 7134 (2143 upregulated, 4991 downregulated), and 9584 (2643 upregulated, 6941 downregulated) DEGs in RM-vs-RL1, RM-vs-RL2, RM-vs-RL3, SM-vs-SL1, SM-vs-SL2, and SM-vs-SL3, respectively, as well as 104, 1108, 745, 141, 280, and 1266 specific genes, respectively. The results indicated that there were more downregulated genes in each comparison group under cold stress. Comparative analysis of six sets of DEGs among the two bitter gourd cultivars yielded 1157 co-expressed genes (Figure 3b).

### 2.5. GO/KEGG Annotation of Cold Stress Genes and Identification of Candidate Gene McNAC087

**Figure 4:**
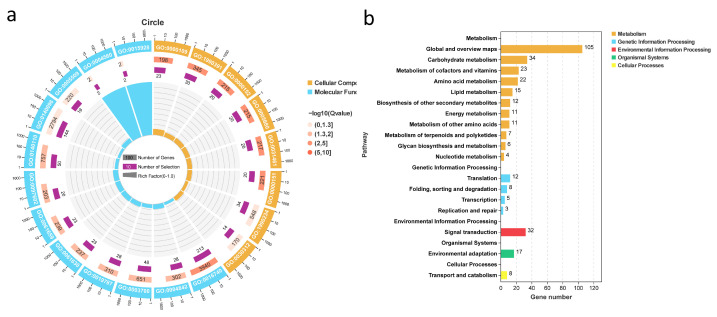
Functional enrichment of DEGs. (**a**) Enriched GO terms in 1157 co-expressed genes. (**b**) Enriched KEGG pathways associated with the 1157 co-expressed genes. The GO term annotations are presented in Table S1.

Gene Ontology (GO) term analysis of the DEGs identified in the six comparison groups revealed their ability to improve the resistance of bitter gourd to low temperatures. The results revealed that 20 significantly enriched terms were annotated and that the DEGs were functionally categorized into cellular component and molecular function terms, but no enriched DEGs were associated with biological process terms. DEGs in the CC category were enriched in nucleotide-excision repair complex (GO:0000109), DNA repair complex (GO:1990391), nuclear ubiquitin ligase complex (GO:0000152), anaphase-promoting complex (GO:0005680), cullin-RING ubiquitin ligase complex (GO:0031461), ubiquitin ligase complex (GO:0000151), transferase complex (GO:1990234), and external encapsulating structure (GO:0030312) terms. The molecular function category included 12 terms, with GO:0016740 (transferase activity), GO:0140096 (catalytic activity), GO:0140110 (protein transcription regulator activity), ubiquitin-like protein transferase activity (GO:0019787), DNA-binding transcription factor activity (GO:0003700), and ubiquitin-protein transferase activity (GO:0004842) being the top 6 terms (Figure 4a).

The main biochemical metabolic pathways and signal transduction pathways associated with the DEGs were identified by significantly enriched metabolic pathways, so we also performed KEGG pathway analysis on the DEGs related to the cold stress response. The top 20 KEGG pathways were divided into five main categories: metabolism, genetic information processing, environmental information processing, organismal systems and cellular processes. Among these pathways, carbohydrate metabolism, signal transduction, amino acid metabolism, and lipid metabolism were closely related to the cold tolerance of bitter gourd (Figure 4b). To date, many *NAC* TFs have been demonstrated to be involved in plant stress responses through direct or indirect regulation of downstream stress-related genes, interactions with other proteins, metabolic regulation, and signal transduction of some hormones [3]. *NAC* TFs play diverse regulatory roles in plant stress responses, and they may regulate the pathways involved in the cold tolerance of bitter gourd. Therefore, we chose *McNAC087* in our co-expressed gene set for functional verification, and experiments were conducted to explore the correlation between *McNAC087* and differential pathways in a follow-up study.

### 2.6. Confirmation of DEGs by RT-qPCR

**Figure 5:**
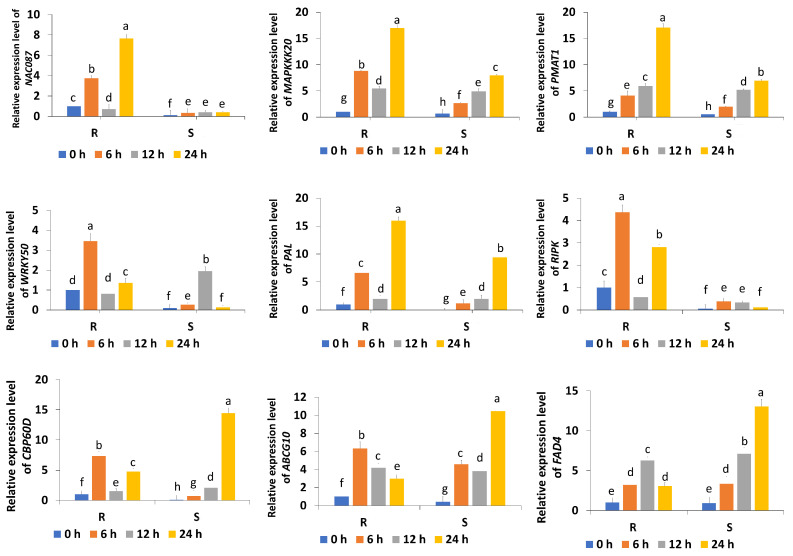
Relative expression levels of genes expressed in response to cold stress, as determined by RT-qPCR. All data are expressed as the means ± standard deviations from three independent experimental replicates. Statistical significance was assessed via analysis of variance (ANOVA), with Duncan’s multiple range test performed subsequently; distinct letters above the bars denote notable differences at *p* < 0.05. R represents the cold-tolerant bitter gourd, and S represents the cold-sensitive bitter gourd. The blue, orange, gray, and yellow bars indicate the relative expression levels at 0 h, 6 h, 12 h, and 24 h of cold stress treatment, respectively.

Nine genes related to cold and environmental stress tolerance were selected, including *NAC087*, *MAPKKK20*, *PMAT1*, *PAL*, *WRKY50*, *CBP60D*, *ABCG10*, *FAD4*, and *RIPK*, from the set of 1157 DEGs. The expression levels of *NAC087*, *PMAT1*, and *RIPK* in R were consistently greater than those in across all cold stress treatment time points (0 h, 6 h, 12 h, and 24 h). The expression levels of *MAPKKK20*, *PAL*, and *WRKY50* were greater in R than in S, especially at 6 h and 24 h of cold stress. In contrast, the expression levels of *ABCG10*, *FAD4*, and *RIPK* peaked in SM after 24 h of cold treatment (Figure 5). The results also confirmed that the general trends of the RNA-seq and RT-qPCR results were consistent.

### 2.7. The Overexpression of McNAC087 Enhances the Cold Resistance of Transgenic Arabidopsis

**Figure 6:**
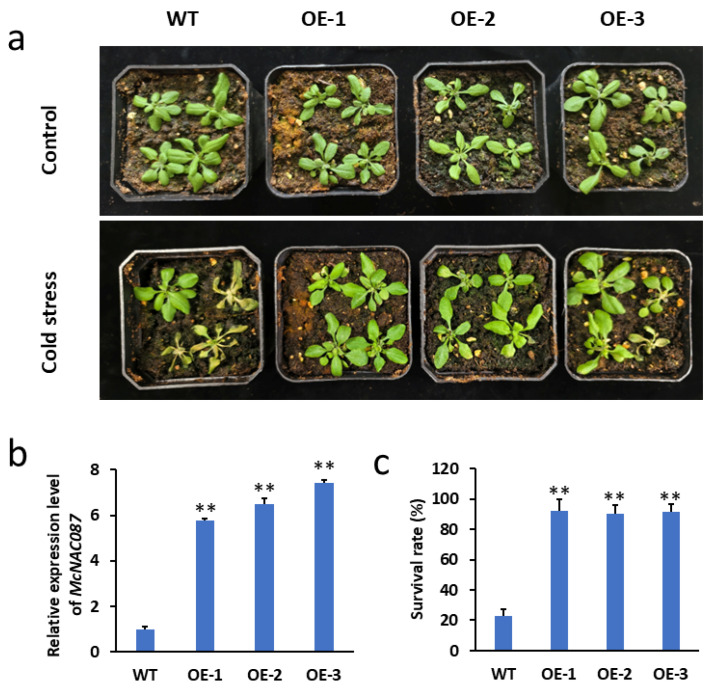
Functional identification of *McNAC087* in transgenic *Arabidopsis thaliana*. (**a**) Phenotypic characteristics of the *McNAC087* transgenic lines and the WT plants under cold stress treatment (−14 °C for 1.5 h, then transferred to 4 °C for 16 h, and finally placed at 22 °C for a 2-day recovery period). Control plants (WT) were kept at 22 °C during the entire experimental period, with no low-temperature treatment applied. (**b**) Relative expression level of *McNAC087* in transgenic *Arabidopsis thaliana*. (**c**) The survival rate of the *McNAC087* transgenic lines and the WT plants under cold stress treatment (−14 °C for 1.5 h, then transferred to 4 °C for 16 h, and finally placed at 22 °C for a 2-day recovery period). Control plants (WT) were kept at 22 °C during the entire experimental period, with no low-temperature treatment applied. Each data reflects the average of three separate experimental repetitions, with error bars denoting the standard errors. Student’s *t* test was employed to evaluate the statistical significance of differences between groups, where ** *p* < 0.01 denotes a highly significant difference.

To investigate the role of McNAC087 in response to low-temperature stress, we constructed the recombinant plasmid pCAMBIA1300-*McNAC087* and transformed it into Arabidopsis using the floral dip technique. The low-temperature stress treatment process involved first exposing four-week-old Col-0 and *McNAC087* overexpressing (OE) lines to −14 °C for 1.5 h, then transferring them to 4 °C for 16 h, and finally placing them at 22 °C for a 2-day recovery period. When grown under ambient conditions, wild-type and transgenic plants showed no apparent differences in morphology; however, the *McNAC087*-overexpressing plants presented a superior growth phenotype, with lower degrees of wilting and yellowing than did the control plants under cold stress treatment (Figure 6a). Moreover, among Arabidopsis plants exhibiting wilting and yellowing symptoms, McNAC087-overexpressing lines exhibited a survival rate varying between 90% and 92%, whereas that of wild-type plants was merely 23% (Figure 6c). Hence, the findings suggested that overexpressing the McNAC087 gene boosted the cold tolerance of the plants.

### 2.8. Overexpression of the McNAC087 Gene Alters Physiological Traits Under Low-Temperature Stress

**Figure 7:**
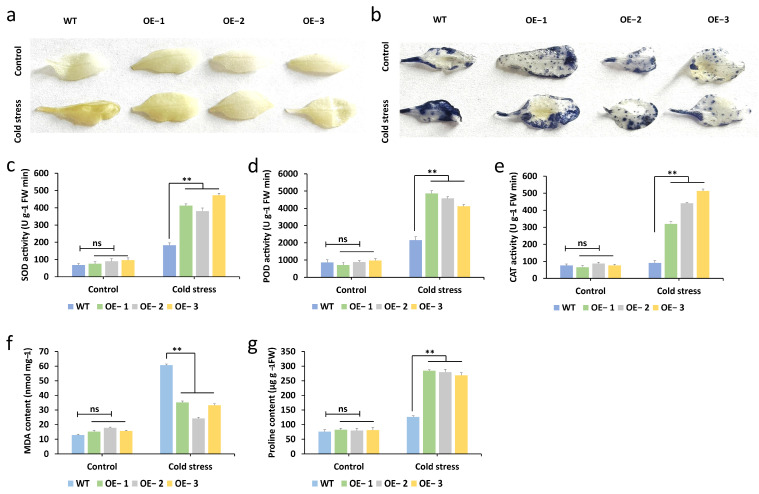
Physiological changes in the *McNAC087* transgenic lines under normal and cold stress conditions. (**a**) DAB staining was used to detect H_2_O_2_. (**b**) NBT staining was used to detect O^2−^. (**c**–**e**) The enzymatic activities of SOD, POD, and CAT in the transgenic lines and WT plants under normal growth conditions and cold stress. (**f**,**g**). The contents of MDA and proline in the transgenic lines and WT plants under normal and cold stress. Each data point represents the mean of three independent experimental replicates, with error bars indicating standard errors. The statistical significance of the intergroup differences was assessed using Student’s *t* test, where ** *p* < 0.01 denotes a highly significant difference, ns indicates non-significant difference.

To gain a deeper understanding of how the *McNAC087* gene influences plant physiological traits under low-temperature stress (4 °C for 24 h), we measured the MDA content, proline content, and activities of SOD, POD, and CAT in Col-0 plants and *McNAC087*-overexpressing lines. The findings revealed that under normal growth conditions, there were no significant differences in proline content, MDA content, or SOD, POD, or CAT activity between the OE lines and Col-0 plants. However, under low-temperature stress, the OE lines presented significantly greater proline contents and antioxidant enzyme activities (POD, SOD, and CAT) than the Col-0 plants did, whereas MDA accumulation showed the opposite trend (Figure 7c–g). Additionally, detection of H_2_O_2_ and O_2_^−^ accumulation via 3,3′-Diaminobenzidine (DAB) and nitroblue tetrazolium (NBT) staining revealed that the OE lines produced less H_2_O_2_ and O_2_^−^ than did the Col-0 plants under low-temperature stress (Figure 7a,b). These findings indicate that *McNAC087* enhances cold stress tolerance by modulating these physiological characteristics.

### 2.9. Analysis of the Expression Patterns of Cold-Responsive Genes in Arabidopsis

**Figure 8:**
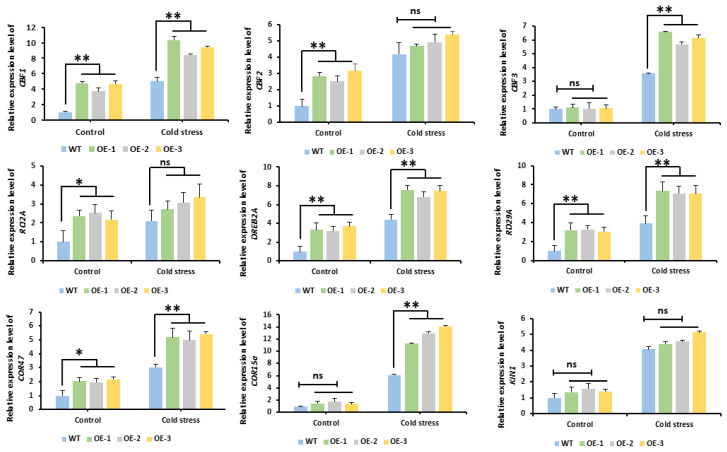
Expression profiling of stress-responsive genes in *Arabidopsis thaliana* exposed to cold stress in wild-type plants and transgenic lines. Each data point signifies the mean of three independent experimental repetitions, with error bars showing standard errors. The statistical significance of differences among groups was determined via Student’s *t* test, where * *p* < 0.05 indicates a statistically significant difference, ** *p* < 0.01 denotes a highly significant difference, and ns indicates non-significant difference.

To explore the potential molecular mechanism by which *McNAC087* regulates cold stress responses, we selected nine stress-responsive marker genes (*CBF1*, *CBF2*, *CBF3*, *RCI2A*, *DREB2A*, *RD29A*, *COR47*, *COR15a*, and *KIN1*) and analyzed their expression levels in Col-0 plants and OE lines under cold stress (4 °C for 24 h). Under normal conditions, the expression levels of *CBF1*, *CBF2*, *RCI2A*, *DREB2A*, *RD29A*, and *COR47* were greater in the overexpression lines than in the Col-0 plants. In contrast, the expression levels of *CBF3*, *COR15a*, and *KIN1* were only weakly correlated with those of *McNAC087*. Moreover, upon cold stress treatment, the expression of all the tested genes in *McNAC087*-overexpressing plants significantly increased. When compared with Col-0 plants, the transcript levels of the *CBF1*, *CBF3*, *DREB2A*, *RD29A*, *COR47*, and *COR15a* genes increased significantly. In summary, these results suggested that the overexpression of the *McNAC087* gene, which improved cold tolerance, may be associated with the expression of these stress-responsive marker genes (Figure 8).

### 2.10. Subcellular Localization of McNAC087

**Figure 9:**
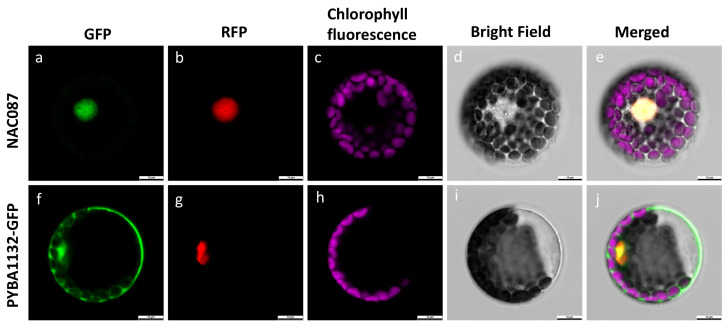
Subcellular localization of McNAC087 (**a**) GFP fluorescence of NAC087; (**b**) RFP fluorescence (nuclear marker) of NAC087; (**c**) Chlorophyll fluorescence of NAC087-expressing cells; (**d**) Bright field image of NAC087-expressing cells; (**e**) Merged image of NAC087, RFP, chlorophyll fluorescence, and bright field. (**f**) GFP fluorescence of pYBA1132-GFP; (**g**) RFP fluorescence (nuclear marker) of pYBA1132-GFP; (**h**) Chlorophyll fluorescence of pYBA1132-GFP-expressing cells; (**i**) Bright field image of pYBA1132-GFP-expressing cells; (**j**) Merged image of pYBA1132-GFP, RFP, chlorophyll fluorescence, and bright field. Scale bars are 10 μm.

To explore the properties of McNAC087, we utilized a transient expression experiment in Arabidopsis protoplasts to characterize where the *McNAC087* protein localizes at the subcellular level. Confocal microscopy revealed that the 35S:GFP fusion protein exhibited diffuse green fluorescence throughout the cell (Figure 9a–e). In contrast, the pYBA1132:McNAC087-GFP fusion protein displayed intense nuclear fluorescence in transformed cells, indicating that McNAC087 localized to the nucleus (Figure 9f–j).

## 3. Discussion

Low temperature is a critical abiotic constraint restricting plant germination, growth, and geographic distribution in modern agricultural production. This phenomenon is particularly pronounced for tropical vegetables cultivated in nontropical regions or other areas prone to cold stress [16]. One of the most fundamental and effective approaches to address this issue lies in breeding cold-tolerant bitter gourd varieties with intrinsic cold resistance. To gain deeper insights into how bitter gourd responds to cold-induced stress, it is crucial to detect cold-induced tolerance-associated genes and dissect their regulatory mechanisms. The *NAC* (*NAM*, *ATAF1/2* and *CUC2*) transcription factor family is the fourth largest TF family in plants and is widely present in various species. NAC proteins participate in nearly all stages of plant growth and development, including cell division, secondary wall formation, shoot apical meristem establishment, floral organ development, fruit ripening, and leaf senescence [3,17]. As plant-specific transcription factors, NAC proteins play pivotal roles in plant responses to abiotic stresses. *CaNAC064* acts as a positive regulator of cold tolerance in pepper plants [18]. The overexpression of *AmNAC24* improved cold and osmotic stress tolerance in *Arabidopsis thaliana*, potentially through the maintenance of ROS homeostasis [19]. *ONAC095* has contrasting regulatory effects on cold and drought stress tolerance in rice; it functions as a negative regulator of drought responses but acts as a positive regulator of cold responses [20]. *PeNAC*-*19* significantly responds to cold stress in tobacco and Arabidopsis, and it can increase cold stress tolerance in yeast [21]. However, to date, not only are functional studies on members of the *NAC* family in bitter gourd scarce, but research examining the biological roles of these family members in the response of bitter gourd to abiotic stresses also remains insufficient. For this study, we performed transcriptome sequencing analyses on the leaves of R and S plants at different time points under cold stress. Through the analysis of core gene sets across six groups of differentially expressed genes—specifically RM-vs-RL1, RM-vs-RL2, RM-vs-RL3, SM-vs-SL1, SM-vs-SL2, and SM-vs-SL3—1157 core genes with potential roles in low-temperature responses were identified. Further functional characterization of these genes revealed that they are involved mainly in carbohydrate metabolism, signal transduction, amino acid metabolism, and lipid metabolism, among other processes. Studies have shown that *NAC* transcription factors can participate in hormone signal transduction to respond to plant stress [3]. We focused on one *NAC* TF, *NAC087*, which indicated that the expression level of *McNAC087* in R was consistently greater than that in SM at 0 h, 6 h, 12 h, and 24 h under cold stress treatment according to RT-qPCR. On the basis of these results, we propose that *NAC087* may be involved in the response to cold stress.

Low temperature represents a major abiotic factor that constrains germination, growth, and distribution [22]. When subjected to cold stress, plants typically undergo various physiological and biochemical alterations while regulating expression of genes to facilitate cold acclimation [23,24]. To safeguard cells against reactive oxygen species (ROS)-induced damage, plants have developed a dedicated antioxidant protection system, which includes antioxidant enzymes and antioxidants, during long-term evolution. In plants, CAT, POD, and SOD are the main antioxidant enzymes. These compounds are able to reduce ROS accumulation and further prevent the peroxidation of membrane lipids, prevent damage to cell structures, and increase plant cold resistance [25]. In addition, when plants are exposed to low-temperature stress, elevated proline levels increase the water retention capacity of cells or tissues, and proline functions as a protectant for enzymes and cellular structures and serves as a carbohydrate source [26]. Moreover, the buildup of H_2_O_2_, O_2_^−^, and MDA can impair the integrity of the plasma membrane and oxidize biological macromolecules in plants [27]. After *JfDREB1A* was introduced into *A. thaliana*, the survival rate of transgenic Arabidopsis plants increased, primarily by preserving cell membrane stability, reducing electrical conductivity, and increasing the activities of antioxidant enzymes such as SOD, POD, and CAT [28]. Moreover, *MbMYBC1* overexpression in Arabidopsis increased the activity levels of CAT, POD and SOD, along with the content of proline, under low-temperature and drought stresses [29]. *VvWRKY28*-overexpressing Arabidopsis lines presented changes in many physiological and biochemical indicators to adapt to cold and high salt stress, including increased activities of SOD, POD, and CAT; increased contents of chlorophyll and proline; and decreased contents of MDA [30]. In the present study, heterologous overexpression of the McNAC087 gene in Arabidopsis enhanced cold tolerance. This increase was likely linked to changes in the following physiological parameters of the *McNAC087* transgenic lines under cold stress: reduced MDA content; decreased H_2_O_2_ and O_2_^−^ levels, increased proline content, and increased POD, SOD, and CAT activities.

Together, the three aforementioned key components, the *ICE*, *CBF*, and *COR* genes, constitute a crucial signaling pathway known as the *ICE*-*CBF*-*COR* cascade. This is a cold-responsive pathway that mitigates cold stress in plants [31]. To further investigate the possible molecular mechanism of the *McNAC087* response to cold stress, the transcription levels of genes related to the *ICE*-*CBF*-*COR* cascade (*CBF1*, *CBF2*, *CBF3*, *RCI2A*, *DREB2A*, *RD29A*, *COR47*, *COR15A* and *KIN1*) were detected via RT-qPCR. Under cold stress, all of these genes were induced under cold stress in both wild-type and transgenic *McNAC087* Arabidopsis. The gene expression of *CBF1*, *DREB2A*, *RD29A* and *COR47* was upregulated in the *McNAC087* overexpression lines compared with the wild type, both under normal and cold conditions. Therefore, *McNAC087* may regulate *Arabidopsis thaliana* tolerance to cold stress by modulating the *ICE*-*CBF*-*COR* pathway.

**Figure 10:**
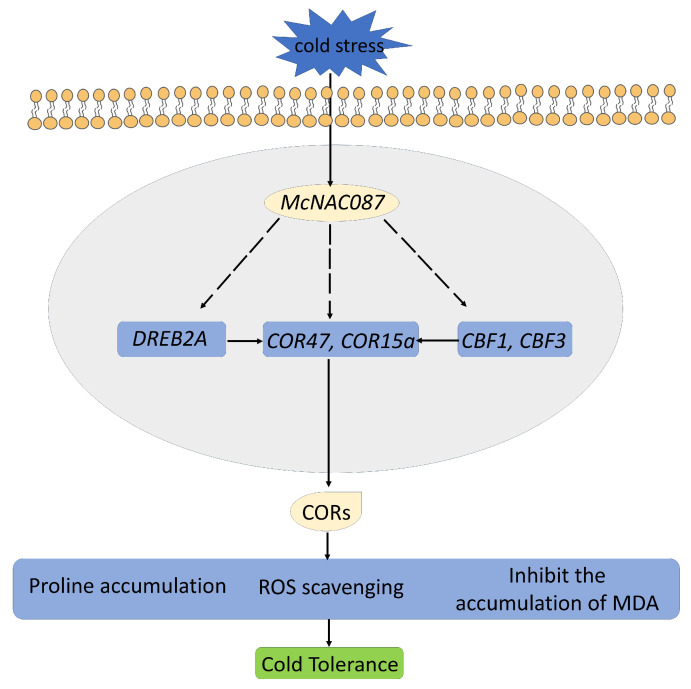
A proposed model illustrating the mechanism of *McNAC087* mediating cold tolerance in plants.

*McNAC087* is activated under cold stress and regulates the expression of downstream target genes, including *DREB2A*, *COR47*, *COR15a*, *CBF1*, and *CBF3*. These regulated genes also promote the expression of COR genes. The latter then promote proline accumulation, enhance ROS scavenging capacity, and inhibit the accumulation of MDA. Through these combined effects, plants achieve higher cold tolerance (Figure 10).

However, the investigation into the cold-responsive characteristics of the bitter gourd *McNAC087* gene in this study was conducted on the basis of overexpressing plants in Arabidopsis. To further clarify the key mechanism of *McNAC087* in the response of bitter gourd to low temperatures, *McNAC087*-overexpressing transgenic bitter gourd materials will be created in the future. Field identification will be carried out to evaluate the growth performance of *McNAC087* transgenic bitter gourd under natural low-temperature conditions. Moreover, various technical approaches, such as transcriptome sequencing, proteome sequencing, and yeast one-hybrid assays, will be employed to analyze in detail the molecular mechanism by which *McNAC087* regulates cold tolerance in bitter gourd. In addition, plans have been made to introduce the *McNAC087* gene identified from bitter gourd into other thermophilic vegetable crops, aiming to explore its potential for improving the cold tolerance of different crops, assess its application value in agricultural production, and contribute to the breeding of cold-tolerant vegetable varieties.

## 4. Materials and Methods

### 4.1. Plant Materials, Growing Conditions, and Cold Stress Treatment

The experimental materials used were bitter gourd inbred lines “0208” (cold stress tolerant, abbreviated as “R”) and “2206” (cold stress sensitive, abbreviated as “S”), provided by the Jiangxi Academy of Agricultural Sciences, China. After soaking in water for 24 h, the seeds were germinated in a 32 °C incubator for 48 h (with 80% relative humidity to prevent seed dehydration), the germinated seeds were sown in seedling trays filled with nutrient soil. The core component of the nutrient soil was commercially available Pindstrup seedling-specific peat (Denmark) (Model: 505+, particle size: 0–6 mm, pH value: 6), which was purchased from a formal horticultural substrate supplier. Subsequently, it was mixed and formulated with other modified substrates at the following volume ratio: 60% Pindstrup peat + 25% perlite + 15% vermiculite. One seed was sown per cell of the tray, and then covered with a 1–2 cm thick layer of nutrient soil. Subsequently, the seedling trays were placed in a growth chamber for cultivation, with the cultivation conditions of 30 °C/28 °C (day/night), 14 h/10 h photoperiod, and 10,000 lx light intensity. When the seedlings reached the four-leaf and one-heart stage, they were subjected to low-temperature treatment (5 °C). The selection of 5 °C as the cold stress temperature was based on previous studies on bitter gourd cold tolerance [16], this temperature is defined as a typical “chilling stress” for bitter gourd seedlings, which can induce obvious cold-responsive phenotypes (e.g., leaf wilting, electrolyte leakage) without causing irreversible cell death. This setting ensures that we can accurately capture the dynamic changes in physiological and molecular responses during the initial phase of cold response, which aligns with the core objective of this experiment: identifying cold-responsive genes by comparing the transcriptome differences between R and S. Leaf samples were collected from R and S at four time points—0 h (before cold stress, control), 6 h, 12 h, and 24 h post-treatment—with three biological replicates per time point. All samples were immediately frozen in liquid nitrogen and stored at −80 °C. For clarity, the samples from R at the above time points were designated as RM (0 h), RL1 (6 h), RL2 (12 h), and RL3 (24 h), respectively; corresponding samples from S were named SM (0 h), SL1 (6 h), SL2 (12 h), and SL3 (24 h), respectively.

Arabidopsis seeds were disinfected three times with 70% alcohol, each time for one minute. Finally, the disinfected Arabidopsis seeds were placed on sterile filter paper. After drying, the seeds were sown in a half MS solid medium (containing 1.5% sucrose) and placed at 4 °C for three days for stratification. Then, they were cultured in a 22 °C incubator (16 h day/8 h night). After Arabidopsis grew two leaves, uniformly sized healthy seedlings were selected and transferred to nutrient soil with substrate/vermiculite/perlite (3:1:1).

### 4.2. RNA Isolation and Transcriptome Sequencing

The total RNA of 24 samples, including RM, RL1, RL2, RL3, SM, SL1, SL2 and SL3 (three biological replicates per material), was extracted using the Plant RNA Kit (Omega Bio-Tek, Norcross, GA, USA) according to the manufacturer’s instructions. To determine the purity of the nucleic acid, a Nanodrop 2000 (Thermo Fisher Scientific, Waltham, MA, USA) was used to determine the OD value. The RNA integrity was determined via an Agilent 2100 (Agilent Technologies, Santa Clara, CA, USA). Finally, the samples were processed by Guangzhou Kidio Biotechnology Co., Ltd. (Guangzhou, China) and sequenced on an Illumina NovaSeq X Plus (Illumina, Inc., San Diego, CA, USA).

### 4.3. Identification of Differentially Expressed Genes and Bioinformatic Analysis

To obtain high-quality clean reads, the sequencing data generated by sequencing machines were further filtered via fastp [32]. Reads with adapter contaminants, more than 10% of nucleotides of undetermined identity, and a low-quality (Q value ≤ 20) base percentage over 50% were removed from subsequent analysis. The short read alignment tool Bowtie2 [33] was employed to conduct the alignment with Ribosome RNA (rRNA). The clean reads were subsequently mapped to the reference genome [34]. To quantify the expression abundance and variations, the fragments per kilobase of transcript per million mapped reads (FPKM) value was computed using RNA-Seq by Expectation Maximization (RSEM) [35]. DESeq2 [36] was subsequently employed to conduct differential expression analysis of the RNAs. The thresholds for screening DEGs were set as FDR < 0.05 and |log2 (fold change)| > 2. To explore the biological functions of the target genes, we used GO (http://www.geneontology.org/ (accessed on 5 April 2025)) to comprehensively describe the properties of genes and their products, as well as KEGG (http://www.genome.jp/kegg/ (accessed on 10 April 2025)), to identify significantly enriched metabolic pathways and signal transduction pathways among the DEGs [37,38].

### 4.4. Arabidopsis Transformation and Cold Stress Tolerance Evaluation

The complete coding sequence of *McNAC087* was amplified from R cDNA using gene-specific primers (Supplementary Table S2) and inserted into the overexpression vector pCAMBIA1302, resulting in the 35S:McNAC087-GFP construct. The recombinant plasmid was subsequently introduced into *Agrobacterium tumefaciens* GV3101 through freeze–thaw technique, and transgenic Arabidopsis plants were generated via the floral dip method. Transgenic plants were selected on 1/2 MS media supplemented with 50 μg/mL hygromycin. Homozygous T3 lines were used for RT-qPCR and phenotype analysis.

To assess cold tolerance, 4-week-old soil-grown transgenic and wild-type (WT) plants were subjected to cold stress at −14 °C for 1.5 h and then transferred to a 4 °C environment for 16 h, followed by a 2-day recovery period at 22 °C to obtain the phenotypes and survival rates. The sequential stress regime (−14 °C → 4 °C → 22 °C) was designed to simulate the “freeze–thaw cycle” that bitter gourd may encounter in natural field conditions (e.g., sudden night frost in early spring followed by gradual temperature rise the next day): the short-term −14 °C treatment (1.5 h) mimics acute freezing stress (to induce cell ice damage), the subsequent 4 °C incubation (16 h) simulates the prolonged low-temperature period after freezing, and the 22 °C recovery step is used to evaluate the plant’s ability to repair freeze damage—this directly matches the objective of verifying whether *McNAC087* overexpression enhances “freezing tolerance” (a key aspect of cold stress). Additionally, in the physiological parameter determination (Section 4.6), we set a 4 °C for 24 h treatment as a “chronic chilling stress control”—this comparison helps distinguish whether *McNAC087* specifically improves freezing tolerance or broadly enhances chilling tolerance, thereby clarifying the functional specificity of the gene. Control plants (WT) were kept at 22 °C during the entire experimental period, with no low-temperature treatment applied. Three independent transgenic lines (OE-1, OE-2, OE-3) and WT plants were included in each experiment. Each line was tested using no fewer than 40 plants, and the experiment was conducted with independent replication at least three times.

### 4.5. RNA Extraction and Quantitative Real-Time PCR Analysis

In order to obtain genes involved in low-temperature resistance, hub DEGs were identified across six comparative groups: RM-vs-RL1, RM-vs-RL2, RM-vs-RL3, SM-vs-SL1, SM-vs-SL2, and SM-vs-SL3. Total RNA samples were isolated using TIANGEN RNA Extraction Kit (Beijing, China) then converted into cDNA via reverse transcription according to Transcriptor First-Strand cDNA Synthesis Kit (Roche, Mannheim, Germany). Totally, eleven genes were selected for RT-qPCR to verify the accuracy of the transcriptome data, and nine stress-responsive marker genes (*CBF1*, *CBF2*, *CBF3*, *RCI2A*, *DREB2A*, *RD29A*, *COR47*, *COR15a*, and *KIN1*) were used to analyze their expression levels in Col-0 plants and OE lines. The primers were designed via Primer 5.0 software (Supplementary Table S2). RT-qPCR was performed on a QuantStudio 7 Flex Real-Time PCR System (Applied Biosystems, Waltham, MA, USA). We assessed the relative gene expression levels according to the 2^−ΔΔCT^ method [39].

### 4.6. Determination of Physiological Parameters

Seeds of WT and *McNAC087*-overexpressing Arabidopsis were grown in soil, and cold stress (4 °C for 24 h) was applied to the plants at 4 weeks. 3,3′-Diaminobenzidine (DAB) and nitroblue tetrazolium (NBT) staining were employed to assess H_2_O_2_ and O_2_^−^ levels. Immerse intact leaves in DAB (pH adjusted to 3.8 with 1 M HCl, freshly prepared before use) and NBT (prepared with 50 mM sodium phosphate buffer, pH 7.8) solution, vacuum infiltrate for 15 min (0.08 MPa) to ensure solution penetration, respectively. After incubating the leaves at 25 °C in the dark for 8 h, destain them with boiling 95% ethanol until chlorophyll is removed. Finally, observe the staining status of the leaves. SOD, POD, and CAT activities, as well as MDA and proline levels, were assayed using spectrophotometric methods. Fresh leaf tissues (0.5 g each) were collected from 4-week-old rosette leaves in transgenic Arabidopsis. The tissues were ground into a homogenate in a pre-cooled mortar with extraction buffer provided in the corresponding kit (tissue:buffer = 1:9, *w*/*v*) under ice-bath conditions. The homogenate was placed in a centrifuge tube, centrifuged at 8000× *g* for 10 min at 4 °C, and the supernatant was subsequently collected to serve as the crude enzyme solution. (for SOD, POD and CAT activity determination) or metabolite extract (for MDA and proline content determination). Repeated freezing and thawing were avoided throughout the process to prevent enzyme activity loss or metabolite degradation. SOD, POD, CAT, MDA and proline were measured using Detection Kit (BC0175, BC0095, BC0205, BC0025 and BC0295; Solarbio, Beijing, China) according to the manufacturer’s instruction.

### 4.7. Subcellular Localization Analysis of McNAC087

The coding sequence CDS of McNAC087 without the stop codon was PCR-amplified and fused to the N-terminus of green fluorescent protein (GFP) in the pYBA1132 vector. The resulting McNAC087:GFP construct was then delivered into Arabidopsis mesophyll protoplasts via polyethylene glycol (PEG)-mediated transfection. Following incubation in the dark for 24 h, the localization results were visualized using a laser scanning confocal microscope (IX83-FV1200, Olympus, Tokyo, Japan).

## 5. Conclusions

In the present study, a total of 1157 co-expressed genes were identified through comparative analysis of DEG sets between two bitter gourd cultivars via RNA sequencing. KEGG analysis indicated that the signal transduction pathway has a close correlation with cold tolerance in bitter gourd, and *NAC* transcription factors may regulate this pathway. The relative expression levels of *McNAC087* in R were consistently greater than those in SM at all cold stress treatment time points, suggesting that *McNAC087* is poised to play a pivotal role in boosting cold tolerance. Subcellular localization analyses revealed that McNAC087 is nuclear-localized. Overexpressing *McNAC087* in Arabidopsis was found to increase cold resistance in transgenic seedlings, as demonstrated by higher proline accumulation, enhanced activities of antioxidant enzymes, elevated antioxidant enzyme activities, and positive regulation of cold-induced gene expression. Collectively, these findings provide valuable insights into the mechanisms underlying cold regulation in bitter gourd.

## Figures and Tables

**Table 1:** Basic statistics of transcriptome sequencing data for bitter gourd seedling samples. Seedlings at the four-leaf and one-heart stage were treated with 5 °C low temperature. Leaf samples were collected from the cold-tolerant line (R) and cold-sensitive line (S) at 4 time points: 0 h (before cold stress, control), 6 h, 12 h, and 24 h post treatment, with 3 biological replicates per time point. Sample naming: R-derived samples = RM (0 h), RL1 (6 h), RL2 (12 h), RL3 (24 h); S-derived samples = SM (0 h), SL1 (6 h), SL2 (12 h), SL3 (24 h); “-1/-2/-3” indicate biological replicates. All base quantities are in bp.

Sample	Total Raw Reads	Total Clean Reads (%)	Total Clean Bases	Clean Reads Q30 (%)	GC Content (%)
RM-1	40,018,692	39,893,848 (99.69%)	5,892,853,624	5,735,816,993 (97.34%)	2,721,951,784 (46.19%)
RM-2	42,835,104	42,663,970 (99.60%)	6,294,026,844	6,122,549,196 (97.28%)	2,904,925,895 (46.15%)
RM-3	39,476,622	39,366,156 (99.72%)	5,810,570,919	5,671,334,032 (97.60%)	2,686,534,839 (46.24%)
RL1-1	40,893,740	40,743,182 (99.63%)	6,022,858,080	5,824,480,038 (96.71%)	2,745,037,914 (45.58%)
RL1-2	48,058,064	47,896,750 (99.66%)	7,073,938,218	6,849,945,058 (96.83%)	3,226,081,644 (45.61%)
RL1-3	39,433,994	39,265,546 (99.57%)	5,830,950,164	5,654,256,539 (96.97%)	2,681,993,642 (46.00%)
RL2-1	37,975,582	37,825,924 (99.61%)	5,597,834,381	5,444,217,924 (97.26%)	2,546,110,109 (45.48%)
RL2-2	43,237,410	43,027,214 (99.51%)	6,367,927,150	6,162,603,972 (96.78%)	2,882,536,185 (45.27%)
RL2-3	42,298,414	42,158,916 (99.67%)	6,270,987,686	6,109,302,688 (97.42%)	2,862,340,959 (45.64%)
RL3-1	41,215,956	41,046,658 (99.59%)	6,039,075,711	5,864,527,892 (97.11%)	2,803,041,196 (46.42%)
RL3-2	36,423,022	36,315,076 (99.70%)	5,346,884,016	5,211,124,486 (97.46%)	2,483,918,080 (46.46%)
RL3-3	45,698,842	45,556,060 (99.69%)	6,708,210,242	6,539,270,593 (97.48%)	3,116,849,459 (46.46%)
SM-1	41,163,672	41,035,338 (99.69%)	6,066,036,632	5,911,629,664 (97.45%)	2,810,644,738 (46.33%)
SM-2	40,644,408	40,503,714 (99.65%)	5,989,237,980	5,836,799,898 (97.45%)	2,777,363,713 (46.37%)
SM-3	38,901,736	38,791,550 (99.72%)	5,733,378,673	5,595,459,985 (97.59%)	2,656,090,816 (46.33%)
SL1-1	44,059,370	43,907,724 (99.66%)	6,508,407,863	6,302,246,629 (96.83%)	2,931,273,138 (45.04%)
SL1-2	43,099,768	42,949,204 (99.65%)	6,353,584,714	6,184,131,820 (97.33%)	2,907,200,197 (45.76%)
SL1-3	39,855,366	39,681,250 (99.56%)	5,881,590,044	5,693,476,583 (96.80%)	2,666,575,152 (45.34%)
SL2-1	42,366,614	42,212,362 (99.64%)	6,236,239,419	6,076,107,478 (97.43%)	2,846,297,902 (45.64%)
SL2-2	44,085,818	43,874,656 (99.52%)	6,486,341,791	6,278,963,701 (96.80%)	2,938,327,609 (45.30%)
SL2-3	43,235,248	43,092,714 (99.67%)	6,380,155,079	6,217,591,803 (97.45%)	2,915,883,323 (45.70%)
SL3-1	45,315,638	45,165,830 (99.67%)	6,686,262,990	6,520,401,992 (97.52%)	3,074,245,023 (45.98%)
SL3-2	38,973,144	38,818,152 (99.60%)	5,753,672,630	5,581,677,280 (97.01%)	2,647,618,786 (46.02%)
SL3-3	40,610,978	40,486,400 (99.69%)	5,970,974,264	5,823,732,363 (97.53%)	2,746,837,661 (46.00%)

## Data Availability

The raw sequencing data generated in this study are available in SRA of NCBI with the accession number PRJNA1331498.

## Supplementary Materials

The following supporting information can be downloaded at: https://www.mdpi.com/article/10.3390/plants14223440/s1, Table S1: GO term annotations; Table S2: List of primers and their uses.

## Abbreviations

The following abbreviations are used in this manuscript:

TF	Transcription factor
COR	Cold-responsive
rRNA	Ribosomal RNA
FPKM	Fragment per kilobase of transcript per million mapped reads
RSEM	RNA-seq by expectation maximization
DEGs	Differentially expressed genes
GO	Gene ontology
WT	Wild-type
DAB	3,3′-diaminobenzidine
NBT	Nitroblue tetrazolium
MDA	Malondialdehyde
POD	Peroxidase
SOD	Superoxide dismutase
CAT	Catalase
GFP	Green fluorescent protein
PEG	Polyethylene glycol
PCA	Principal Component Analysis
ROS	Reactive oxygen species
