# Supplementary material for: The Bitter Gourd Transcription Factor McNAC087 Confers Cold Resistance in Transgenic Arabidopsis
Source: Plants (Basel). 2025 Nov 10;14(22):3440. doi: 10.3390/plants14223440 (PMC12656278; doi:10.3390/plants14223440)
Supplement: Supplementary file 1 [file plants-14-03440-s001.zip › supplementary file/Table S2.pdf]

Table S2 List of primers and their uses.

Primer	Sequence	Used for
OE-*NAC087*-F	acacgctgacaagctgactctagcagatctATGGAAGAGCTACCGGCGA	Overexpression
OE-*NAC087*-R	aaaagttcttctcctttgcccatggctctagaaTCAGTAATCCCACAGGTCCA	
S-*NAC087*-F	caattggagctccaccgcggtggcggccgcATGGAAGAGCTACCGGCGACCGCCGTGGAGTAC	Subcellular localization
S-*NAC087*-R	gctcctcgcccttgctcaccatggtaccGTAATCCCACAGGTCCAGATCCGCC	
q-*NAC087*-F	ATTTAGATTCCACCCTACCGACG	qRT-PCR
q-*NAC087*-R	CACTTGTTCAAGTCGGCTTCT	
q-*MAPKKK20*-F	GGACATGAAATTGGACATGGCA	
q-*MAPKKK20*-R	CTTCTCGTTCTTAAGCGAGGAAG	
q-*PMAT1*-F	ACCACCCTCACCTAATTGAAGT	
q-*PMAT1*-R	CGCCGTTTGATTATCGGGTAT	
q-*PAL*-F	CCTTCAAAAGGAGCTCATCCG	
q-*PAL*-R	TCACCAACATGGCTGCTCT	
q-*WRKY50*-F	TACGTCTGAGAGCGATCTTGC	
q-*WRKY50*-R	TAAACCGGGTACATGACCGA	
q-*CBP60D*-F	CAATGGGGTCAGATTTCGCG	
q-*CBP60D* -R	TTCAAGAGGCTGAAATTGCCG	
q-*ABCG10*-F	ATGGACTTGCCAATGAAGAGG	
q-*ABCG10*-R	CCACCACACAGCCAATTCAA	
q-*FAD4*-F	GAACCCGTGTTAACCCGTTG	
q-*FAD4*-R	GCCCAATGTGAGTGGAGGAGA	
q-*RIPK*-F	AATGGCGTGGAAATCGATGAT	
q-*RIPK*-R	GCTTTTCTTCGTAACCCGCC	
q-*CBF1*-F	TGTCTCAACTTCGCTGACTCGGC	
q-*CBF1*-R	ACCTTCGCTCTGTTCCGGTGTATAA	
q-*CBF2*-F	GGTTTCCTCAGGCGGTGATTACAGT	
q-*CBF2*-R	TCAGCGGTTTGGAAAGTCCCGAGCC	
q-*CBF3*-F	TATTTCAGCAAACCATACCAAC	
q-*CBF3*-R	CTCTAACCTCACAAACCCACTT	
q-*RCI2A*-F	ATCGCCATCCTCTTGCCTCC	
q-*RCI2A*-R	TAGGAGAACACGACGGAAC	
q-*DREB2A*-F	GGTAAAGGAGGACCAGAGAATAGCC	
q-*DREB2A*-R	AGACGAGCCAAAGGACCATACATAG	
q-*RD29A*-F	GTCTGCCGTGACGACGAAGTTAC	
q-*RD29A*-R	TCCTTCTTCTCTTCTTCTCCTCCAA	
q-*COR47*-F	TATCATGCCAAGACCACTGAA	
q-*COR47*-R	CAACGAAAGCCACAATAACAA	

Primer	Sequence	Used for
q-*COR15a*-F	GGTAAAGCAGGAGAGGCTAAGGATG	
q-*COR15a*-R	AAGAATGTGACGGTGACTGTGGATA	
q-*KIN1*-F	GGACCAACAAGAATGCCTTCCAAGC	
q-*KIN1*-R	CGCTGCCGCATCCGATACACT	
MCActin 7-F	CCCTCCCTCATGCAATTCTC	Internal controls in bitter gourd
MCActin 7-R	GTTACATGTTTACCACTACTGCCGA	
AtActin-F	GCACCAAGCAGCATGAAGA	Internal controls in Arabidopsis
AtActin-F	GAACCACCGATCCAGACACT	
